# Recent Advances and Future Challenges in the Additive Manufacturing of Hydrogels

**DOI:** 10.3390/polym14030494

**Published:** 2022-01-26

**Authors:** Chris Danek

**Affiliations:** 1 Bessel LLC, San Carlos, CA 94070, USA; cjdanek@utep.edu; 2 Mechanical Engineering Department, W.M. Keck Center for 3D Innovation, The University of Texas at El Paso, El Paso, TX 79968, USA

## 1. Introduction

The emergence of additive manufacturing, otherwise known as 3D printing, was predated by significant advances in the understanding and controlled engineering of hydrogels.

Hydrogels swell or contract dramatically as a function of their water content. They may be formulated to respond based on environmental factors, such as temperature, pH, mechanical or electrical field stimulation, ion or solvent concentrations or light stimulation [1,2,3]. Based on this behavior, hydrogels may be configured as sensors for these environmental stimuli [4,5]. Alternatively, environmental stimulus factors may be used as control signals for hydrogels designed as actuators. Early practical applications were proposed before the emergence of additive manufacturing. Some of these early examples included potential use as artificial muscles or microfluidic flow control devices [6,7]. Hydrogels also offer the potential for a chemically active function, either in aqueous solution or by functionalizing the polymer hydrogel itself. As an example, glucose introduced into a hydrogel by iontophoresis was used in combination with an enzyme to create a glucose sensor [8].

Additive manufacturing refers to a layer-by-layer creation of parts from a digital file. Additive manufacturing brings the ability to create complex, three dimensional geometries with hydrogels and to vary material composition throughout fabricated parts. Additive manufacturing of hydrogels typically involves liquid precursors, which are cross linked, layer by layer. This makes it easy to incorporate multiple materials of varied types during fabrication. Additive manufacturing process categories that are commonly used with hydrogel materials include vat photopolymerization by stereolithography (SL) or digital light processing (DLP) and material extrusion by direct ink writing [9].

The potential benefits of hydrogels and the unique capabilities of additive manufacturing made additive manufacturing of hydrogels an early, advanced application area for additive manufacturing. Hydrogels received only two minor mentions in an early (1993) review of tissue engineering—for use in corneal tissue generation and as microcapsules for cell transplant [10]. By 1998, additive manufacturing of hydrogel scaffolds for tissue engineering was demonstrated [11]. Soon after, 3D robotic dispensing systems based on syringe deposition were developed and used to create hydrogel scaffolds for tissue engineering [12,13,14]. Around the same time, researchers used photolithography to create three-dimensional hydrogel scaffolds containing living cells [15].

The community has made substantial progress in the additive manufacture of hydrogels over the past two decades. There are a number of excellent reviews of this progress, including on these topics:

- Comprehensive reviews of materials, processes and biomedical applications [16,17,18,19];
- Focused review of tissue engineering applications and strategies for biofabrication of artificial organs and organ-on-a-chip technology [20,21,22];
- Soft robotics, smart materials and smart structures, including shape memory and self-healing materials, and complex, origami-inspired structures [23,24,25,26];
- Hydrogel materials: hydrogels with functionalized polymers; hydrogel/reactive filler/embedded cell bioinks; bioinks with nano-biomaterials for tissue engineering; and biomaterials [27,28,29,30];
- Bioresorbable electronics [31];
- Multi-material additive manufacturing, with soft robotics and biomedical applications [32].

Below are select recent advances toward compelling hydrogel applications, developed at least in part through additive manufacturing processes. Despite these advances, many of the potential applications will not be a long-term fit for additive manufacturing. Drawing on experience from other additive manufacturing application areas, the author proposes criteria for use cases that will be a good fit for additive manufacturing.

## 2. Recent Advances in Additive Manufacturing of Hydrogels

There are exciting advances promising the incorporation of hydrogels into functional designs in areas such as soft robotics and organ-on-a-chip technology. Many of these advances have been enabled by additive manufacturing processes ***as a research tool***. The pace of discovery in this field is accelerating. Mechanical performance improvements include a wider range of control of the stress–strain relationships through hydrogel network composition and the creation of hydrogel materials that break the prior links between mechanical strength and parameters such as fatigue performance and recovery response time. There have been significant advances in the hybrid fabrication of hydrogel–polymer composite structures, as well as in the performance and reliability of hydrogel-based sensors and actuators.

### 2.1. Improved Mechanical Performance for Biomedical and Soft Robotic Applications

Highly stretchable hydrogel structures may be covalently bonded with ultraviolet-cured polymers by a digital light processing (DLP) vat photopolymerization additive manufacturing process [33]. This process is attractive because it gives designers the freedom to finely control three-dimensional stiffness gradients and to independently harness the unique properties of hydrogels for sensing, drug elution or mechanical response to the environment. The speed and resolution of this process is an advance over previous investigators that created similar hydrogel–polymer systems by material extrusion [34]. Another promising application of these covalently bonded hydrogel–polymer structures is for soft robotics: constrained-layer hydrogel flexures can be designed to contract when the hydrogel constituents swell [35].

Additive manufacturing will certainly continue to put the latest improvements in hydrogel mechanical properties to full use. Dual-hydrogel networks significantly improve toughness and can be leveraged to create self-healing hydrogel structures and hydrogel structures engineered to strengthen on repeated stress [36]. Researchers recently demonstrated strong, tough and fast-recovering hydrogels by including zinc–peptide-based metal coordination complexes into the hydrogel network [37]. Additive manufacturing can combine multi-material and gradient fabrication to take full advantage of the wider range of mechanical properties available with advanced hydrogels.

### 2.2. Advances in Additive Manufacturing of Hydrogels for Sensors, Actuators and Soft Robotics 

Researchers developed an autonomous, light-powered and oscillation-propelled hydrogel robot suitable for additive manufacturing, along with a model for the robot’s performance to tune oscillation frequency and locomotive force at varied robot sizes [38]. Another non-invasive method to control soft robots is the magnetic actuation of hydrogels with embedded nano-magnetic particles [39]. By incorporating DNA sequences into hydrogel structures, the CAS enzyme used in CRISPR can be used to selectively cleave the hydrogel, for applications in cell and drug release, sensing and actuation [40]. Synthetic biology has been applied to hydrogel-based robotics: engineered cells converted biological sensing to fluorescence, with the optical signal used for feedback control of a hydrogel actuator [41]. Traditional manufacturing methods created two-dimensional gradients in acoustic impedance by embedding steel cylinders in hydrogel for broadband acoustic impedance matching, useful for ultrasonic sensing [42]. Additive manufacturing is able to give additional control over three-dimensional acoustic impedance gradients, with the promise of corresponding advances in acoustic sensors.

### 2.3. Improvements in Additive Manufacturing of Hydrogels for Tissue Engineering

Vascularization of tissue grown on scaffolds is a key challenge. Researchers recently demonstrated the potential feasibility of DLP-based vat photopolymerization additive manufacturing of hydrogels with interconnected microchannels for use as synthetic skin grafts [43]. The scientific community is working toward the engineering of artificial cells [44]. A future step will be to include artificial cells in the additive manufacture of organ-on-a-chip constructs.

## 3. The Role of Additive Manufacturing with Hydrogels: Where Does It Fit?

The ability to rapidly fabricate complex geometries makes additive manufacturing a great tool for rapid iteration in design and research. Where might additive manufacturing excel for production part fabrication? The most compelling use case is the additive manufacturing of hydrogel parts with composition gradients. In many cases, production processes through traditional methods can be developed to produce parts that are designed with the help of additive manufacturing [45]. This holds, even though additive manufacturing is well suited to parts with (internal) geometric complexity, is capable of multi-material fabrication and can produce integrated multi-functional parts (for example, with embedded electronics) [46].

Typically, after a design is proven with rapid prototyping tools, it will be transferred to manufacturing using traditional processes for cost-effective production. This is the case as well for hydrogel applications. Traditional methods of hydrogel manufacture include laminating sheets of hydrogel or casting hydrogel in place [47,48]. Hydrogels may be cast around complex structures for increased functionality [49]. The advances in multi-material additive manufacturing may be largely replaced by multistep traditional manufacturing methods, for example, by casting hydrogel materials of varying composition in multiple steps using investment casting methods or casting directly in an injection-molded plastic part [48].

Injection-molded parts with laminated films are cost-effective manufacturing processes for lab-on-a-chip designs using traditional polymer materials [50]. Similar complexity with hydrogel materials is available by including hydrogels cast in place in these multi-material designs. The lines blur somewhat with automated fluid dispensing and curing equipment. However, even with robotic dispensing of the hydrogel, the process bears more similarity to the automated dispensing of adhesives or gaskets in traditional manufacturing because the hydrogel is not built layer by layer.

Hydrogel scaffolds for tissue engineering and organ-on-a-chip applications are arguably more advanced than the use of hydrogels for soft robotics. These biomedical applications make good test cases for where additive manufacturing best contributes beyond rapid prototyping. Sacrificial molding is a common approach to incorporating hydrogels with complex internal geometries into organ-on-a-chip devices [44,48]. The challenge of creating long, small-diameter lumens in hydrogel tubing to mimic vascular transport may also be addressed by micromolding of hydrogels with lumens with diameter of 250 μm [51]. Two-step casting processes may thus be used to create ‘vascularized’ hydrogel tissue engineering constructs. Such methods obviate the clear need for additive manufacturing.

Traditional manufacturing methods using multi-step fabrication thus offers a significant cost advantage over the additive manufacturing of hydrogels in most situations. Based on the compliant nature of hydrogels, it is hard to argue for mass customization of most hydrogel-centric design applications. Other than use cases that suggest a need for ‘manufacture on demand’ of hydrogel devices using additive manufacturing, the primary remaining long-term advantage that additive manufacturing holds over traditional processes is for designs that rely on the gradient composition manufacture to achieve highly localized performance. This would include designs for hydrogels, such as those already highlighted, to enable localized actuation, sensing and drug delivery [38,39,40,41].

Until applications are developed that use the localized performance achieved through gradient composition manufacture, we should expect that the primary role for additive manufacturing of hydrogels will continue to be for rapid prototyping of research test beds and design candidates.

## Data Availability

Not applicable.
